# Supplementary material for: Quantitating SARS-CoV-2 neutralizing antibodies from human dried blood spots
Source: Microbiol Spectr. 2024 Oct 29;12(12):e00846-24. doi: 10.1128/spectrum.00846-24 (PMC11619372; doi:10.1128/spectrum.00846-24)
Supplement: Supplemental material — Tables S1 to S4; Fig. S1 to S4. [file spectrum.00846-24-s0001.pdf]

Table S1. SARS-CoV-3 Reagents and Sources.				
**Reagent**	**Description**	**Source**	**Catalogue Number**	**Usage**
TBS	Tris Buffered Saline with Casein	BioRad	1610782	DBS Elution
FLT	Full Length Trimeric SARS-CoV-2 Spike	MassBiologics	N/A	8-Plex MIA/iACE2
FLS	Full length SARS-CoV-2 Spike, His-Tag	Native Antigen	REC31868-100	8-Plex MIA
Spike Subunit 1 (S1)	SARS-CoV-2 (2019-nCoV) Spike S1, His-Tag	Sino Biological	40591-V08H	8-Plex MIA
RBD	SARS-CoV-2 Stable RBD, Thrombin-His	MassBiologics	N/A	8-Plex MIA
Nucleocapsid (N)	2019-nCoV Nucleocapsid Protein, His tag	SinoBiological	40588-V08B	8-Plex MIA
Nucleocapsid (N-NA)	SARS-CoV-2 Nucleoprotein, His-Tag (E. coli)	Native Antigen	REC31812-100	8-Plex MIA
Nucleocapsid (NHT)	SARS-CoV-2 (2019-nCoV) Nucleocapsid Protein (His tag)	Sino Biological	0588-V07E	8-Plex MIA
Internal Control (IC)	Mouse anti-Human IgG3 Secondary Antibody	ThermoFisher	MA1-83242	8-Plex MIA
WT sRBD	SARS (WA1/2020) RBD, Thrombin-His	MassBiologics	N/A	iACE2
hACE2	Biotinylated ACE2	Wadsworth Center Protein Core	N/A	iACE2
CC12.3	Human Mab RBD-A WT D614G	Scripps	N/A	iACE2/RVP
RVP-702L	WT D614G Strain Reporter Virus Particles	Integral Molecular	CL-275A	RVP
293-hsACE2 Cells	293T cells expressing human ACE2 receptor	Integral Molecular	TA-060520-MC	RVP

Table S2. Sensitivity Comparison of 8-plex MIA with Abbott Advise Dx SARS-CoV-2 IgG II assay.

Assay	8-plex MIA				AdviseDx SARS-CoV-2 IgG II
Sample Type	cDBS				Plasma
Sample	S1 (MFI)	FLS (MFI)	TRI (MFI)	RBD (MFI)	Result (AU/ml)
A 1:1	7,506	11,046	13,695	12,136	2,791.9
A 1:2	3,644	7,113	9,097	9,035	1,315.4
A 1:4	2,542	4,086	6,361	6,414	637.7
A 1:8	1,190	2,370	3,180	3,305	323.7
A 1:16	773	1,267	1,871	1,864	169.5
A 1:32	412	772	1,052	1,292	85.7
A 1:64	243	434	633	714	46.6
A 1:128	172	202	394	569	28.7
A 1:256	78	114	285	488	19.4
A 1:512	111	64	231	441	14.8
B 1:1	11,524	19,433	20,260	19,134	19,537.8
B 1:2	7,540	15,573	15,959	18,859	9,395.8
B 1:4	8,318	13,237	12,448	14,998	4,218.9
B 1:8	4,841	7,609	7,755	9,658	1,998.8
B 1:16	2,983	5,249	5,225	6,257	947.3
B 1:32	1,589	3,082	3,298	3,976	464.0
B 1:64	1,075	2,189	2,125	2,706	239.0
B 1:128	516	933	1,124	1,252	124.6
B 1:256	365	548	657	1,119	66.3
B 1:512	173	275	407	621	40.0
C 1:1	4,415	13,194	18,733	12,508	4,761.0
C 1:2	3,578	10,327	15,306	10,729	2,174.7
C 1:4	1,396	4,527	8,413	5,638	1,008.9
C 1:8	1,022	3,600	6,374	4,102	478.5
C 1:16	490	1,775	3,380	2,365	246.7
C 1:32	239	1,001	2,189	1,270	126.0
C 1:64	169	502	1,194	799	71.9
C 1:128	110	238	533	509	39.0
C 1:256	104	136	330	421	23.0
C 1:512	104	86	231	321	16.0

Three positive plasma samples were two-fold serially diluted (1:1 through 1:512) and tested on the Abbott assay. The positive plasma was also combined with equal parts blood cells and spotted onto DBS cards to create paired DBS. The DBS were tested with the 8-plex MIA. Spike MIA reactivity levels were similar to the Abbott assay. Red, yellow and green shading indicates reactive, indeterminate and negative results, respectively, on each assay.

Table S3. Seroconversion Panel Sensitivity.

	8-plex MIA MFI						
**# Days post first blood draw**	**N**	**N (NA)**	**NHT**	**S1**	**FLS**	**TRI**	**RBD**
1	104	157	55	82	15	69	54
3	86	149	60	66	23	68	57
10	101	160	72	63	20	68	49
15	73	152	42	67	23	62	48
17	82	134	55	59	15	81	54
24	84	127	65	59	13	68	58
31	77	111	70	61	19	56	51
36	90	210	68	73	173	1,005	139
50	1,503	4,935	883	649	1,877	6,185	2,080
64	2,775	6,200	1,375	1,607	3,050	7,909	4,077
71	2,818	5,421	1,227	1,433	2,723	6,664	3,816
78	2,631	5,440	1,392	1,325	2,627	6,856	3,440
80	2,793	5,887	1,626	1,417	2,811	7,502	3,825
87	1,560	3,797	840	819	1,692	4,456	2,026

A seroconversion panel (Access Biologicals) was tested with the 8-plex MIA. FLS and TRI showed positivity earlier than the other antigens. The inclusion of FLS and TRI makes the 8-plex assay more sensitive than the previous 2-plex assay. Red, yellow, and green shading indicates reactive, indeterminate, and negative results, respectively, on each assay.

Table S4. Comparison of NAB-Sure™ Inhibition (%) values in Panel D and Panel E cDBS eluates

	Inhibition (%)	
**Dilution (1/x)**	**Panel D**	**Panel E**
2*	73 +/-16.9	25 +/-13.8
6*	45 +/-19.6	12 +/-5.3
18	22 +/-12.9	10 +/-6.1
54	11 +/-8.0	6 +/-7.5
162	9 +/-4.2	16 +/-10.9
486	14 +/-8.7	8 +/-7.2

Panel D and Panel E were evaluated using the NAB-Sure™ assay. Panel D represents the average (+/- SD) of the 15 samples and Panel E is the average of 7 samples. Inhibition (%) was compared between the two groups. Asterisks indicate a difference between groups as determined by 2-way ANOVA (* indicates $p<0.0001$).

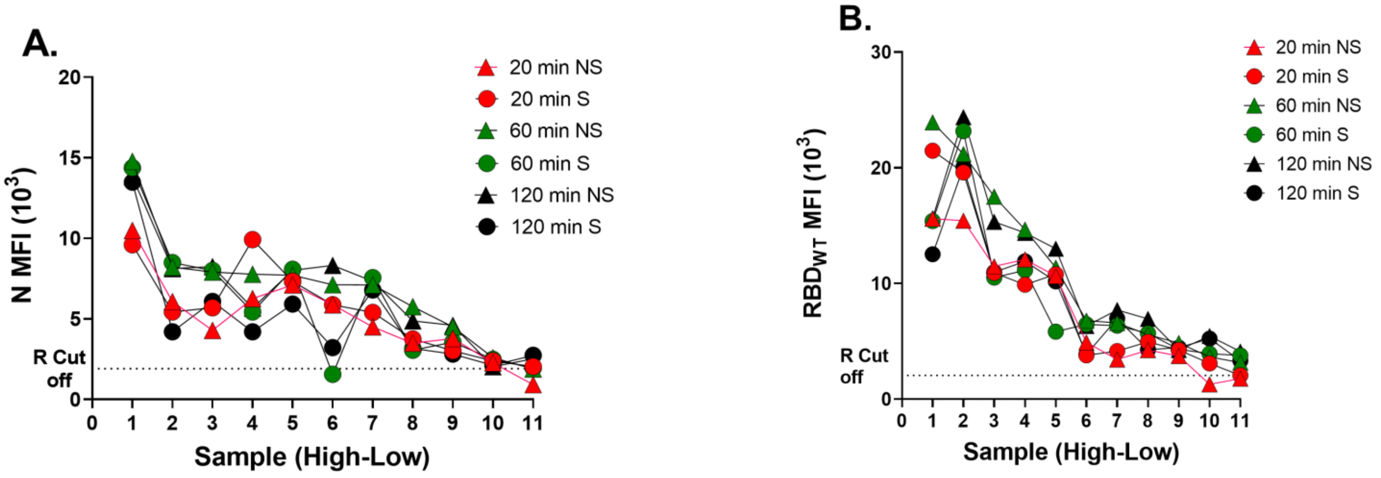

Figure S1. DBS Elution Optimization. Nine Panel D and 2 positive control DBS were eluted for the following times: 20 minutes, 60 minutes, and 120 minutes. Each time point was also tested with shaking (S) at 300 RPM and no shaking (NS). DBS eluates were rank ordered from high to low (left to right) based on N MFI **(A)** and RBD MFI **(B).** 20 minutes proved too short of an elution time as some positive samples did not cross the reactive cutoff. Minimal MFI differences were noted between 60 minutes and 120 minutes. The optimal elution condition that produced the highest MFI is 60 minutes no shaking. The dotted lines represent the reactive cutoff for N (1,908) and RBD (2,049). Negative Control DBS were also eluted and tested (data not shown).

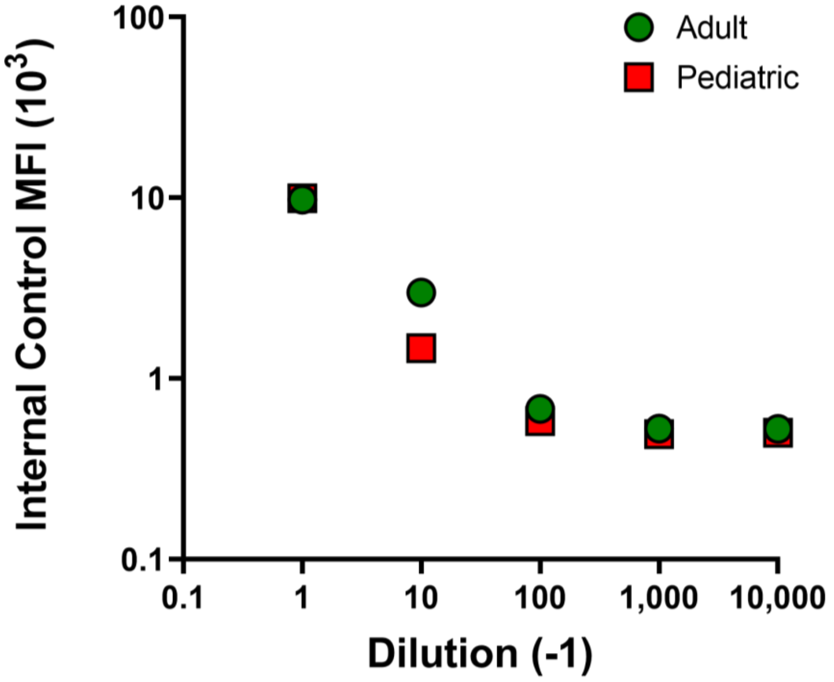

Figure S2. Internal Control. Five adult and five pediatric DBS samples were 10-fold serially diluted and tested with the internal control bead (mouse anti-human IgG3). MFI of all 10 samples decreased as sample became more dilute, showing that the assay is sensitive to changes in the amount of sample added to the well. One representative from the adult and pediatric samples are graphed.

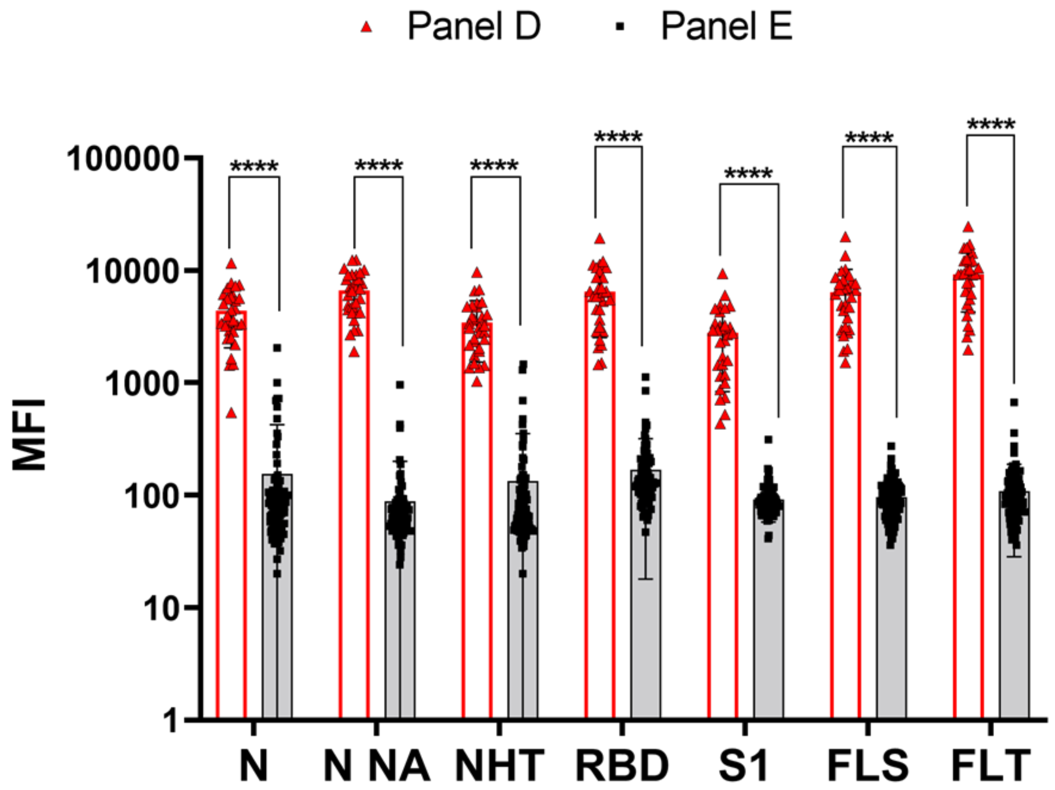

Figure S3. Comparison of Panel D and Panel E cDBS in MIA. The full cohort for Panel D (n=30) and Panel E (n=86) samples underwent testing via 8-plex MIA. MFI values for the following antigens are compared: N, N NA, NHT, RBD, S1, FLS and FLT. Asterisks indicate a significant difference between groups by 2-way ANOVA, where ****p<0.0001.

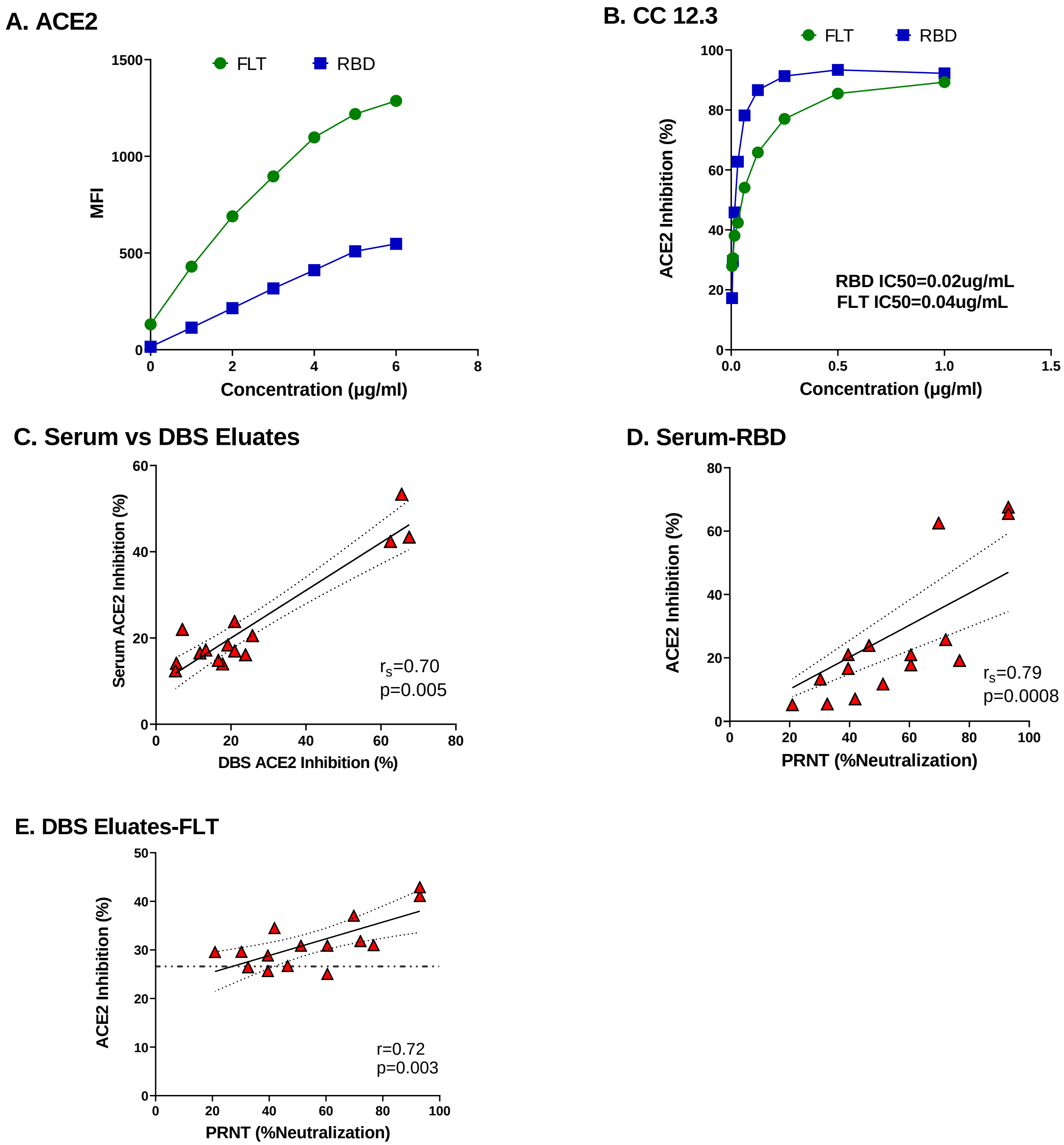

Figure S4. Optimization of Luminex-based ACE2 inhibition assay. (A) MFI values (binding) was assessed in the context of either FLT or RBD with varying concentrations of biotinylated hACE2. **(B)** ACE2 inhibition titers associated with CC 12.3 in the context of FLT or RBD. IC_{50} is shown as text. **(C)** Spearman correlation of Panel D serum samples and DBS eluates analyzed by ACE2 inhibition, r_s-value and p-value shown as text. **(D)** Spearman correlation of Panel D serum analyzed by ACE2 inhibition and SARS-CoV-2 neutralizing activity (%), r_s-value and p-value shown as text. **(E)** Pearson correlation of Panel D DBS eluates (n=15) analyzed by ACE2 inhibition and SARS-CoV-2 neutralizing activity (%), in the context of FLT. The r-value and p-value are shown as text. The dotted line on **(E)** represents the average iACE2 activity for SARS-CoV-2 negative cohort (Panel E; n=7).
